# Fixation of Intertrochanteric Fractures: Dynamic Hip Screw versus Locking Compression Plate

**DOI:** 10.5812/traumamon.10436

**Published:** 2013-08-11

**Authors:** Mohsen Mardani-Kivi, Ahmadreza Mirbolook, Sina Khajeh Jahromi, Melina Rouhi Rad

**Affiliations:** 1 Orthopedic Department of Pursina Hospital, Guilan University of Medical Science, Rasht, IR Iran; 2 Student Research Committee, Guilan University of Medical Sciences, Rasht, IR Iran

**Keywords:** Hip Fractures, Infection, Joint, Fracture Fixation

## Abstract

**Background:**

According to the existing literature, the Dynamic Hip Screw (DHS) is the preferred standard for the treatment of intertrochanteric fractures. However, some surgeons use other devices such as the Locking Compression Plate (LCP).

**Objectives:**

In this study, we compared the outcome of using DHS or LCP in intertrochanteric fractures.

**Materials and Methods:**

This cross-sectional study was carried out on 104 patients who were referred to Pursina Hospital in Rasht, Iran with intertrochanteric fractures of the femur treated with either the DHS or LCP devices. Demographic features, existence or nonexistence of stability and operating time were obtained from questionnaires. During a 6-month follow-up after surgery, patients were interviewed to record variables such as Harris Hip Scores and complications. The patients were also interviewed on their final visit (between 9 and 31 postoperative months). The collected data was analyzed using SPSS.

**Results:**

We discovered that the number of incidences of limb shortening and device failure was higher for patients treated with the LCP device (P = 0.048 and P = 0.014). Patients treated with the DHS device had higher Harris Hip scores for both the 6-month postoperative and the final evaluation visits (P = 0.01 and P = 0.018).

**Conclusions:**

Despite the complications of fixation with the DHS device, it remains the most successful for treatment of intertrochanteric fractures.

## 1. Background

More than 90% of hip fractures in patients after the 5th decade of age are intertrochanteric fractures with 20-30% of these cases experiencing complications and a mortality rate of approximately 17% (1-3). Intertrochanteric fractures of the femur occur between the greater trochanter, the attachment site to the hip abductor and extensor muscles, and the lesser trochanter, the attachment site of the hip flexor muscle (3). In the elderly, these fractures typically result from mild to moderate trauma due to osteoporotic bones while in young adults, these fractures are generally due to high energy trauma, such as road accidents (2). The incidence of hip fractures is 2-3 times more common in females and the risk of fracture will double, every 10 years after the age of 50 (4). Operative treatment is the best option in most cases of hip fractures (5). There are several devices that may be used for fracture fixation. The Dynamic Hip Screw (DHS) is a screw that allows for controlled dynamic sliding of the femoral head and is used to fix both the femoral head and the device to the shaft of the femur. The dynamic compression allows the weight-bearing stresses to stabilize the femur so that it may undergo remodeling and proper fracture healing. After 30 weeks, 75% of the patients had their normal function restored (6). Although this device is suggested as the gold standard for the treatment of fractures of the proximal femur, there are now various new devices for fracture fixation (3). One such new device is the Locking Compression Plate (LCP), an implant plate with a stable angle for management of comminuted and osteoporotic fractures. The LCP is stated to be more suitable for stable and osteoporotic intertrochanteric fractures (7-9).

## 2. Objectives

Although the DHS device has been the treatment of choice for intertrochanteric fractures, the use of the LCP device by some surgeons has warranted an examination of the effectiveness of these methods (1, 3, 10). In this study, treatment results using the LCP and DHS devices on intertrochanteric fractures were compared.

## 3. Materials and Methods

This cross-sectional study of patients with trochanteric fractures of the femur that were treated with either the DHS or LCP device at Pursina Hospital in Rasht, Iran from March 2009 until 2011 was conducted. There were 54 patients who had polytraumatic or pathologic fractures; patients with previous surgery in this same anatomical region and DJD in the hip joint were excluded. Other patients, who were never available for follow-ups, due to death or other reasons, were also excluded. A total of 104 patients were included in this study and all patients’ information was considered highly confidential.To select the patients we took into account various factors such as the availability of the device, the economic situation of the patients and the environment of the operating room. All patients underwent an operation by one orthopedic specialist (the first author of the paper). After general anesthesia and reduction under fluoroscopy, patients were prepared for the fracture fixation with a DHS or LCP device through a lateral approach. The location of the nail in the fixation using the DHS device and the screw in the fixation using the LCP device was determined by radiography. For approximately 48 hours, a drain was used. Patients were discharged when they had partial weight bearing ability on the fracture. All patients were evaluated for rotation of the femur with the patella in a horizontal position. Demographic features such as age and gender, the existence or nonexistence of fracture stability (comminuted fractures owing displacement of lesser trochanter, with posterior medial defect and reverse oblique fractures are unstable fractures) (10) and operating time were obtained via questionnaire. During both the 6-month postoperative visit and the final visit (between 9 and 31 months after the surgery), patients were examined regarding certain variables such as the Harris Hip Score (to evaluate the function of hip joint) and existence of common complications, including limb shortening (in the following visits), device failure and infection. A diminished measurement of more than 20 mm was considered as limb shortening, cutting out or breaking of the device was considered as device failure and serous or purulent discharge from the incision site was considered as evidence of an infection. Analysis was performed by descriptive statistics, the Chi square test and independent t-test. All data was analyzed by SPSS software (P < 0.05 was considered statistically significant).

## 4. Results

Of the 104 patients in this study, 69 were males (66.3%) and 35 were females (33.7%). Sixty patients with a mean age of 74.6 years were treated with the DHS device and 44 patients with a mean age of 73.1 years were treated with the LCP device. There were no significant differences among the age and gender of these two groups. The demographic features are briefly shown in Table 1. Of the 104 patients in this study, 39 patients (37.5%) had stable fractures and 65 patients (62.5%) had unstable fractures. This difference was not significant (Table 2). In the aggregate, 13 patients had device failure, 8 patients had limb shortening and 2 patients developed deep infection. Our results revealed that the incidence of limb shortening and device failure were more in patients treated with the LCP device (P = 0.048 and P = 0.014).The fixation time using the DHS device was less than with the LCP device (P < 0.0001, Table 1).

**Table 1.** Comparison of Demographic Features and Operating Time between Two Group of DHS and LCP

	DHS	LCP	P value
**Gender**			
Female, No. (%)	18 (30)	17 (38.6)	0.35
Male, No. (%)	42 (70)	27 (61.4)	
**Age, Mean ±SD**	74.35 ± 11.18	71.00 ± 10.28	0.11
**Operating time, Mean ±SD, min**	51.33 ± 8.72	71.02 ± 8.99	< 0.0001

**Table 2.** Evaluation of the Implant and Harris Hip Scores on the Basis of Fracture Stability

Fracture Pattern	Implant	No. (%)	P value	Harris Score	P value
**Stable**	DHS	24 (23.1)	0.39	87.08 ± 4.13	0.39
	LCP	14 (13.5)		85.43 ± 7.65	
**Unstable**	DHS	36 (34.6)		84.61 ± 11.45	0.18
	LCP	30 (28.8)		81.20 ± 6.86	

Complications are summarized in Table 3. The Harris Hip Score obtained during the 6-month postoperative visit for patients treated with the DHS device (total of 60 patients) showed that 19 patients (31.7%) had excellent scores, 38 patients (63.3%) had good scores and 3 patients (5.0%) had fair scores. Patients treated with the LCP device (total of 44 patients), 9 patients (20.5%) had excellent scores, 22 patients (50.0%) had good scores and 13 patients (29.5%) had fair scores. The mean scores showed that the difference between patients treated with the DHS or LCP device after 6 postoperative months was significant (P < 0.0001) however, differences between these two groups during the final visit were not statistically significant.

**Table 3.** Complications of the Procedures

Complications	Data	P value
**Device failure, No. (%)**		
DHS	3 (5)	0.014
LCP	10 (22.7)	
**Limb shortening, No. (%)**		
DHS	2 (3.4)	0.048
LCP	6 (13.6)	
**Deep infection, No. (%)**		
DHS	1 (1.6)	0.699
LCP	1 (2.27)	
**Harris hip score, Mean ± SD**		
Six months post operation		0.01
DHS	86.54 ± 5.64	
LCP	82.94 ± 7.39	
Last visit		0.018
DHS	88.04 ± 7.51	
LCP	84.06 ± 7.75	

## 5. Discussion

Nearly half of all hip fractures are intertrochanteric fractures. Even though fixation with the DHS device has been the gold standard treatment for stable intertrochanteric fractures (11, 12), there are many complications reported for unstable intertrochanteric fractures (3-26 %) (10). The therapeutic results of intertrochanteric fractures fixation with LCP have not yet been fully determined. In the study performed by Nordin et al. on intertrochanteric fractures treated with the DHS device, the incidence of device failure was reported to be 16.7% (13); however our study found a lower rate (5%). In comparison, Yong et al.(10) reported that the mean operating time was 74 minutes, the mean Harris Hip Score was 80, the rate of limb shortening was 29% and there was no detection of deep infections; in the present study, the incidence of limb shortening and the rate of operating time were lower; the mean Harris Hip Score was higher however the incidence of deep infection was greater. On the other hand, Ehlinger et al. reported that about 6% of patients with intertrochanteric fractures treated with the DHS device had infections; however no implant loosening was seen (14). In the study of intertrochanteric fractures treated with the LCP device by Yuming et al., the mean operating time was 53.2 minutes, Harris Hip Scores were “excellent” (53.5%), “good” (37.5%), “fair” (6.5%) and “poor” (2.5%) and no infection or limb shortening was reported (15). In the present study, the incidence of infection was less than referenced studies and the rate of Harris Hip Score of “good” was higher. In fixation with the DHS device, varus collapse (whether primary or secondary) and failure of femoral head screw were the most frequent complications reported(16-19). In our study, the incidences of limb shortening and device failure were lower in patients treated with the DHS however the incidence of deep infection was equal in both groups. The mean Harris Hip Score was higher in the patients treated with the DHS device, both at the 6-month postoperative examination and at the final visit. Previous studies produced various results: some reported an improvement for patients with fixation using the LCP device; some reported less complications with fixation using the LCP (20), and others stated that the DHS was the safer device (5, 21).In general, device failure is due to several factors including the type of fracture and its stability, osteoporosis and the incorrect placement of the screw into the femoral head. In the present study, the majority of patients were elderly females with no significant difference in the procedure and the Harris Hip Scores. The operation duration also was different between these two groups. However, proper exercise and rehabilitation of the patients is important (22-24). It seems the existence of several factors can cause different outcomes for these two devices and more studies are needed to determine these factors. Some reports suggest that fixation with the DHS device is preferable; the placement of screw near the subchondral bone can improve fixation (12, 25)and the associated compression to weight bearing aids in the healing of the fracture (26). Other reports propose that fixation with the LCP device is better because placement of the plate is more adaptable for the surgeon and it reduces the deformity of flexion or extension. The plate is inserted through the skin with reduced morbidity (27).In this study, both at the 6-month postoperative examination and the final visit, patients treated with the DHS device had higher Harris Hip Scores. On the basis of this score which shows the qualitative improvement of the hip joint function, we believe the DHS device is preferred over the LCP device. While Zhu et al. and Luo et al. did not report any significant differences between the two groups (28, 29) and in another survey, patients treated with percutaneous compression plate had higher scores when compared with DHS (30), we suggest that the therapeutic results do not merely depend solely on the device utilized. Various factors including the type of fracture and related complications and experience can affect the function of hip joint as demonstrated using the Harris Hip Score.

Despite some concomitant complications, including device failure, infection and limb shortening, use of the DHS device remains the preferred treatment for intertrochanteric fractures.
